# Methyl (2′*S*,3′*S*)-3,4-*O*-(2′,3′-dimethoxy­butane-2′,3′-di­yl)-α-l-rhamnopyran­oside: a glycosyl acceptor

**DOI:** 10.1107/S1600536808008222

**Published:** 2008-04-23

**Authors:** Yow-Fu Tsai, Jen-Ta Yang, Jhy-Der Chen, Chia-Her Lin

**Affiliations:** a Department of Chemistry, Chung-Yuan Christian University, Chung-Li 320, Taiwan

## Abstract

The title compound, C_13_H_24_O_7_, is the product of the ketalization of methyl l-(+)-rhamnopyran­oside with 2,3-butane­dione. It crystallizes with two mol­ecules in the asymmetric unit, which are connected by O—H⋯O hydrogen bonds. The C-3,4 diequatorial hydroxy groups of the methyl l-(+)-rhamnopyran­oside were protected, leaving the C-2 hydroxy group free. The l-(+)-rhamnopyran­oside and 2′,3′-dimethoxy­butane-2′,3′-diyl rings adopt chair conformations and all meth­oxy groups are in axial positions. The absolute configuration was assumed from the synthesis.

## Related literature

For related literature, see: Duynstee *et al.* (1998 ▶); Lang & Wullbrandt (1999 ▶); Leisinger & Margraff (1979 ▶); Montchamp *et al.* (1996 ▶); Bauer *et al.* (2006 ▶).
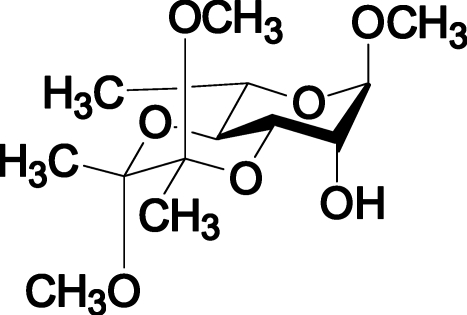


## Experimental

### 

#### Crystal data

C_13_H_24_O_7_
*M* *_r_* = 292.32
Orthorhombic, 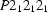

*a* = 12.8743 (14) Å
*b* = 13.1182 (12) Å
*c* = 18.208 (3) Å
*V* = 3075.0 (7) Å^3^
*Z* = 8
Mo *K*α radiation
μ = 0.10 mm^−1^
*T* = 295 (2) K
0.6 × 0.5 × 0.4 mm

#### Data collection

Bruker *P*4 diffractometer
Absorption correction: ψ scan (North *et al.*, 1968 ▶) *T*_min_ = 0.933, *T*_max_ = 0.994
3842 measured reflections
3032 independent reflections
2630 reflections with *I* > 2s(*I*)
*R*_int_ = 0.020
3 standard reflections every 97 reflections intensity decay: none

#### Refinement

*R*[*F* ^2^ > 2σ(*F* ^2^)] = 0.038
*wR*(*F* ^2^) = 0.106
*S* = 1.01
3032 reflections
362 parameters
H-atom parameters constrained
Δρ_max_ = 0.15 e Å^−3^
Δρ_min_ = −0.17 e Å^−3^

### 

Data collection: *XSCANS* (Siemens, 1995 ▶); cell refinement: *XSCANS*; data reduction: *SHELXTL* (Sheldrick, 2008 ▶); program(s) used to solve structure: *SHELXS97* (Sheldrick, 2008 ▶); program(s) used to refine structure: *SHELXL97* (Sheldrick, 2008 ▶); molecular graphics: *SHELXTL*; software used to prepare material for publication: *SHELXTL*.

## Supplementary Material

Crystal structure: contains datablocks I. DOI: 10.1107/S1600536808008222/bt2683sup1.cif

Structure factors: contains datablocks I. DOI: 10.1107/S1600536808008222/bt2683Isup2.hkl

Additional supplementary materials:  crystallographic information; 3D view; checkCIF report

## Figures and Tables

**Table 1:** Hydrogen-bond geometry (Å, °)

*D*—H⋯*A*	*D*—H	H⋯*A*	*D*⋯*A*	*D*—H⋯*A*
O5—H5*A*⋯O13^i^	0.82	2.17	2.907 (3)	150
O12—H12*A*⋯O5^ii^	0.82	2.10	2.846 (3)	152

Symmetry codes: (i) 
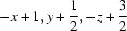
; (ii) 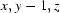.

**Fig. 1.**
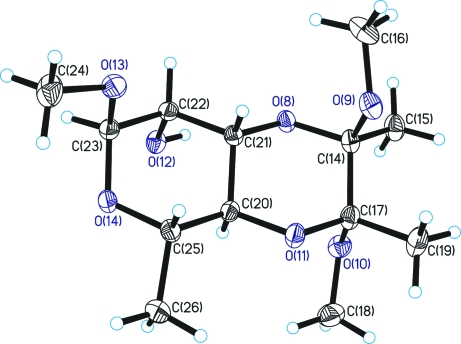
The molecular structure of the title compound, showing the atom numbering scheme. Displacement ellipsoids for non-H atoms are represented at the 30% probability level. The H atoms are drawn with an arbitrary radius.

## supplementary crystallographic information

### Comment

*L*-Rhamnopyranse-containing glycolipids display a wide range of biological properties (Leisinger, & Margraff, 1979; Lang & Wullbrandt, 1999; Bauer *et al.*, 2006). For the above reason, we were interesting to study on the total synthesis of rhamnolipids. The title compound, C_13_H_24_O_7_, which was synthesized by the ketalization of C-3,4 hydroxyl group of the methyl *L*-(+)-rhamnopyranoside, with 2,3-butanedione and chemoselectively protected the C-3,4 diequatorial hydroxyl group (Montchamp *et al.*, 1996; Duynstee *et al.*, 1998), was utilized as glycosyl acceptor in our synthetic strategy. The methyl *L*-(+)-rhamnopyranoside was prepared by acetalization of the commercial optical pure *L*-(+)-rhamnopyranose as starting material with methanol. The structure of *L*-rhamnopyranoside ring and 2',3'-dimethoxybutan-2',3'-diyl ring are chair conformation and all of methoxy groups are at axial position.

### Experimental

A solution of methyl *L*-(+)-rhamnopyranose ([*a*]^20^_D_ = + 8.2°) (638 mg, 3.58 mmol), trimeyhyl orthoformate (1.20 ml, 10.75 mmol) and 2,3-butanedione (0.35 ml, 3.98 mmol) in dried methanol (15 ml) was treated with camphersulfonic acid (50 mg, 0.22 mmol). The mixture was refluxed for 18 h. The cool reaction mixture was then treated with NEt_3_ and concentrated under reduced pressure to observe crude product. The crude product was purified *via* flash column chromatography on silica gel (EtOAc/*n*-hexane = 1:1) to obtain 874 mg (84%) of the title compound as white powder. The pure product was recrystalized from CH_2_Cl_2_ at room temperature.

### Refinement

In the absence of anomalous scatterers Friedel pairs were merged prior to refinement. The C-bound H atoms were placed in calculated positions (C—H = 0.96–0.98 Å) and included in the refinement in the riding-model approximation, with *U*_iso_(H) = 1.2 or 1.5*U*_eq_(C). The hydroxy H atoms were constrained to ideal geometries with O(N)—H = 0.82 Å and *U*_iso_(H) = 1.5*U*_eq_(O).

### Crystal data

**Table:**

C_13_H_24_O_7_	*F*_000_ = 1264
*M**_r_* = 292.32	*D*_x_ = 1.263 Mg m^−^^3^
Orthorhombic, *P*2_1_2_1_2_1_	Mo *K*α radiation λ = 0.71073 Å
Hall symbol: P 2ac 2ab	Cell parameters from 27 reflections
*a* = 12.8743 (14) Å	θ = 5.1–12.5º
*b* = 13.1182 (12) Å	µ = 0.10 mm^−^^1^
*c* = 18.208 (3) Å	*T* = 295 (2) K
*V* = 3075.0 (7) Å^3^	Block, colourless
*Z* = 8	0.6 × 0.5 × 0.4 mm

### Data collection

**Table:**

Bruker P4 diffractometer	*R*_int_ = 0.020
Radiation source: fine-focus sealed tube	θ_max_ = 25.0º
Monochromator: graphite	θ_min_ = 1.9º
*T* = 295(2) K	*h* = −1→15
ω scans	*k* = −1→15
Absorption correction: ψ scan(North *et al.*, 1968)	*l* = −1→21
*T*_min_ = 0.933, *T*_max_ = 0.994	3 standard reflections
3842 measured reflections	every 97 reflections
3032 independent reflections	intensity decay: none
2630 reflections with *I* > 2s(*I*)	

### Refinement

**Table:**

Refinement on *F*^2^	Hydrogen site location: inferred from neighbouring sites
Least-squares matrix: full	H-atom parameters constrained
*R*[*F*^2^ > 2σ(*F*^2^)] = 0.038	*w* = 1/[σ^2^(*F*_o_^2^) + (0.0561*P*)^2^ + 0.7995*P*] where *P* = (*F*_o_^2^ + 2*F*_c_^2^)/3
*wR*(*F*^2^) = 0.106	(Δ/σ)_max_ < 0.001
*S* = 1.02	Δρ_max_ = 0.15 e Å^−^^3^
3032 reflections	Δρ_min_ = −0.17 e Å^−^^3^
362 parameters	Extinction correction: SHELXL97 (Sheldrick, 2008), Fc^*^=kFc[1+0.001xFc^2^λ^3^/sin(2θ)]^-1/4^
Primary atom site location: structure-invariant direct methods	Extinction coefficient: 0.0063 (6)
Secondary atom site location: difference Fourier map	

### Special details

**Table:**

Geometry. All e.s.d.'s (except the e.s.d. in the dihedral angle between two l.s. planes) are estimated using the full covariance matrix. The cell e.s.d.'s are taken into account individually in the estimation of e.s.d.'s in distances, angles and torsion angles; correlations between e.s.d.'s in cell parameters are only used when they are defined by crystal symmetry. An approximate (isotropic) treatment of cell e.s.d.'s is used for estimating e.s.d.'s involving l.s. planes.
Refinement. Refinement of *F*^2^ against ALL reflections. The weighted *R*-factor *wR* and goodness of fit *S* are based on *F*^2^, conventional *R*-factors *R* are based on *F*, with *F* set to zero for negative *F*^2^. The threshold expression of *F*^2^ > σ(*F*^2^) is used only for calculating *R*-factors(gt) *etc*. and is not relevant to the choice of reflections for refinement. *R*-factors based on *F*^2^ are statistically about twice as large as those based on *F*, and *R*- factors based on ALL data will be even larger.

### Fractional atomic coordinates and isotropic or equivalent isotropic displacement parameters (Å^2^)

**Table:**

	*x*	*y*	*z*	*U*_iso_*/*U*_eq_	
O1	0.29481 (18)	0.87985 (15)	0.88054 (10)	0.0505 (5)	
O2	0.1553 (2)	0.8215 (2)	0.95033 (13)	0.0673 (7)	
O3	0.35887 (18)	0.66974 (18)	0.87880 (14)	0.0614 (6)	
O4	0.20071 (17)	0.70426 (15)	0.82239 (11)	0.0469 (5)	
O5	0.38039 (16)	0.96048 (16)	0.74810 (11)	0.0502 (5)	
H5A	0.4138	0.9481	0.7854	0.075*	
O6	0.11031 (18)	0.99268 (18)	0.71133 (13)	0.0626 (6)	
O7	0.21798 (18)	0.86713 (17)	0.66048 (11)	0.0529 (5)	
O8	0.38389 (15)	0.21315 (15)	0.83116 (10)	0.0426 (4)	
O9	0.38270 (19)	0.33878 (17)	0.92121 (12)	0.0564 (6)	
O10	0.17861 (17)	0.16879 (17)	0.87757 (12)	0.0548 (5)	
O11	0.20194 (17)	0.33159 (15)	0.82917 (10)	0.0470 (5)	
O12	0.37676 (17)	0.15454 (15)	0.67829 (11)	0.0481 (5)	
H12A	0.3881	0.1110	0.7096	0.072*	
O13	0.43863 (16)	0.41769 (15)	0.66158 (12)	0.0515 (5)	
O14	0.27898 (16)	0.34098 (16)	0.63634 (10)	0.0451 (5)	
C1	0.2596 (3)	0.8024 (3)	0.92943 (17)	0.0570 (9)	
C2	0.3345 (4)	0.8045 (3)	0.99348 (19)	0.0829 (13)	
H2A	0.3352	0.8714	1.0148	0.124*	
H2B	0.4030	0.7875	0.9766	0.124*	
H2C	0.3129	0.7558	1.0298	0.124*	
C3	0.1350 (4)	0.9160 (3)	0.9869 (2)	0.0932 (15)	
H3A	0.0622	0.9210	0.9977	0.140*	
H3B	0.1551	0.9716	0.9557	0.140*	
H3C	0.1740	0.9188	1.0317	0.140*	
C4	0.2534 (3)	0.6969 (2)	0.89081 (17)	0.0507 (8)	
C5	0.3743 (3)	0.5765 (3)	0.8392 (3)	0.0899 (15)	
H5B	0.4473	0.5641	0.8335	0.135*	
H5C	0.3425	0.5817	0.7916	0.135*	
H5D	0.3433	0.5211	0.8659	0.135*	
C6	0.1952 (3)	0.6187 (3)	0.9353 (2)	0.0671 (10)	
H6A	0.1933	0.5553	0.9089	0.101*	
H6B	0.1256	0.6420	0.9438	0.101*	
H6C	0.2297	0.6088	0.9815	0.101*	
C7	0.2429 (2)	0.7820 (2)	0.77618 (15)	0.0426 (7)	
H7A	0.3160	0.7670	0.7659	0.051*	
C8	0.2344 (2)	0.8829 (2)	0.81437 (15)	0.0436 (7)	
H8A	0.1615	0.8952	0.8271	0.052*	
C9	0.2721 (2)	0.9686 (2)	0.76624 (16)	0.0438 (7)	
H9A	0.2589	1.0340	0.7904	0.053*	
C10	0.2127 (3)	0.9639 (2)	0.69401 (17)	0.0497 (7)	
H10A	0.2420	1.0145	0.6603	0.060*	
C11	0.0474 (3)	1.0130 (4)	0.6492 (3)	0.1003 (16)	
H11A	−0.0209	1.0325	0.6651	0.150*	
H11B	0.0428	0.9529	0.6193	0.150*	
H11C	0.0776	1.0674	0.6211	0.150*	
C12	0.1820 (3)	0.7835 (2)	0.70469 (16)	0.0477 (7)	
H12B	0.1080	0.7924	0.7154	0.057*	
C13	0.1975 (3)	0.6876 (3)	0.6604 (2)	0.0698 (10)	
H13A	0.1575	0.6917	0.6160	0.105*	
H13B	0.1751	0.6298	0.6886	0.105*	
H13C	0.2697	0.6802	0.6484	0.105*	
C14	0.3400 (3)	0.2447 (2)	0.89914 (15)	0.0445 (7)	
C15	0.3620 (3)	0.1598 (3)	0.95361 (17)	0.0591 (9)	
H15A	0.4356	0.1497	0.9575	0.089*	
H15B	0.3297	0.0980	0.9370	0.089*	
H15C	0.3345	0.1780	1.0008	0.089*	
C16	0.4938 (3)	0.3455 (3)	0.9215 (2)	0.0743 (11)	
H16A	0.5144	0.4122	0.9375	0.111*	
H16B	0.5197	0.3335	0.8728	0.111*	
H16C	0.5216	0.2952	0.9544	0.111*	
C17	0.2219 (3)	0.2663 (2)	0.89001 (16)	0.0471 (7)	
C18	0.0701 (3)	0.1652 (3)	0.8604 (2)	0.0737 (11)	
H18A	0.0493	0.0956	0.8532	0.111*	
H18B	0.0574	0.2032	0.8162	0.111*	
H18C	0.0310	0.1943	0.9000	0.111*	
C19	0.1755 (3)	0.3204 (3)	0.95614 (18)	0.0642 (10)	
H19A	0.1028	0.3319	0.9479	0.096*	
H19B	0.2099	0.3845	0.9632	0.096*	
H19C	0.1844	0.2788	0.9991	0.096*	
C20	0.2480 (2)	0.2952 (2)	0.76220 (15)	0.0400 (6)	
H20A	0.2181	0.2290	0.7489	0.048*	
C21	0.3632 (2)	0.2850 (2)	0.77310 (14)	0.0388 (6)	
H21A	0.3917	0.3515	0.7869	0.047*	
C22	0.4147 (2)	0.2498 (2)	0.70284 (15)	0.0400 (6)	
H22A	0.4902	0.2467	0.7094	0.048*	
C23	0.3874 (2)	0.3265 (2)	0.64348 (15)	0.0425 (6)	
H23A	0.4147	0.3017	0.5965	0.051*	
C24	0.4307 (3)	0.4943 (3)	0.6062 (2)	0.0701 (10)	
H24A	0.4665	0.5546	0.6222	0.105*	
H24B	0.3589	0.5101	0.5978	0.105*	
H24C	0.4615	0.4699	0.5616	0.105*	
C25	0.2258 (2)	0.3727 (2)	0.70230 (15)	0.0430 (7)	
H25A	0.2511	0.4399	0.7175	0.052*	
C26	0.1112 (3)	0.3797 (3)	0.6822 (2)	0.0652 (10)	
H26A	0.1019	0.4304	0.6447	0.098*	
H26B	0.0718	0.3984	0.7249	0.098*	
H26C	0.0877	0.3149	0.6643	0.098*	

### Atomic displacement parameters (Å^2^)

**Table:**

	*U*^11^	*U*^22^	*U*^33^	*U*^12^	*U*^13^	*U*^23^
O1	0.0683 (14)	0.0485 (11)	0.0347 (10)	−0.0162 (11)	−0.0075 (10)	0.0047 (9)
O2	0.0860 (18)	0.0663 (15)	0.0495 (12)	−0.0112 (14)	0.0156 (12)	−0.0026 (12)
O3	0.0551 (14)	0.0524 (12)	0.0768 (15)	−0.0051 (11)	−0.0090 (12)	0.0158 (12)
O4	0.0527 (12)	0.0444 (10)	0.0436 (10)	−0.0123 (10)	−0.0050 (10)	0.0060 (9)
O5	0.0487 (12)	0.0539 (12)	0.0480 (11)	−0.0057 (10)	−0.0044 (10)	0.0100 (10)
O6	0.0520 (13)	0.0695 (15)	0.0662 (14)	0.0071 (12)	−0.0070 (12)	0.0189 (12)
O7	0.0619 (13)	0.0616 (13)	0.0353 (10)	−0.0092 (11)	−0.0043 (10)	0.0041 (10)
O8	0.0487 (11)	0.0449 (10)	0.0341 (9)	0.0091 (9)	−0.0015 (9)	0.0017 (9)
O9	0.0694 (15)	0.0526 (12)	0.0473 (11)	0.0022 (12)	−0.0088 (11)	−0.0066 (11)
O10	0.0536 (12)	0.0520 (12)	0.0587 (13)	0.0000 (11)	0.0034 (11)	0.0075 (11)
O11	0.0538 (12)	0.0503 (11)	0.0368 (9)	0.0139 (10)	0.0053 (9)	0.0036 (9)
O12	0.0582 (12)	0.0424 (10)	0.0436 (10)	−0.0060 (10)	0.0036 (10)	−0.0028 (9)
O13	0.0543 (12)	0.0473 (11)	0.0530 (12)	−0.0147 (10)	−0.0035 (11)	0.0055 (10)
O14	0.0481 (12)	0.0549 (12)	0.0323 (9)	−0.0046 (10)	−0.0023 (8)	0.0026 (9)
C1	0.071 (2)	0.062 (2)	0.0384 (15)	−0.0189 (18)	−0.0021 (16)	0.0082 (14)
C2	0.119 (3)	0.086 (3)	0.0434 (18)	−0.035 (3)	−0.025 (2)	0.0195 (19)
C3	0.131 (4)	0.087 (3)	0.062 (2)	−0.003 (3)	0.024 (3)	−0.012 (2)
C4	0.0558 (19)	0.0491 (17)	0.0472 (16)	−0.0094 (15)	−0.0059 (15)	0.0139 (14)
C5	0.069 (2)	0.0473 (19)	0.154 (5)	0.0036 (19)	0.001 (3)	0.009 (3)
C6	0.077 (2)	0.063 (2)	0.062 (2)	−0.025 (2)	−0.009 (2)	0.0213 (17)
C7	0.0476 (16)	0.0417 (14)	0.0384 (14)	−0.0063 (13)	−0.0001 (13)	0.0035 (13)
C8	0.0511 (17)	0.0457 (15)	0.0342 (14)	−0.0060 (13)	−0.0045 (13)	0.0027 (13)
C9	0.0492 (16)	0.0419 (15)	0.0401 (15)	−0.0013 (13)	−0.0001 (13)	0.0040 (12)
C10	0.0528 (18)	0.0503 (17)	0.0460 (16)	−0.0033 (15)	−0.0022 (15)	0.0133 (14)
C11	0.066 (3)	0.142 (4)	0.093 (3)	0.003 (3)	−0.024 (2)	0.051 (3)
C12	0.0505 (17)	0.0518 (16)	0.0409 (15)	−0.0078 (15)	−0.0031 (14)	0.0011 (14)
C13	0.085 (3)	0.069 (2)	0.056 (2)	−0.006 (2)	−0.010 (2)	−0.0167 (18)
C14	0.0571 (18)	0.0438 (15)	0.0327 (14)	0.0051 (14)	−0.0022 (13)	−0.0014 (12)
C15	0.077 (2)	0.0559 (18)	0.0441 (16)	0.0182 (18)	−0.0011 (16)	0.0077 (15)
C16	0.074 (2)	0.075 (2)	0.074 (2)	−0.008 (2)	−0.031 (2)	0.000 (2)
C17	0.0574 (18)	0.0465 (16)	0.0373 (14)	0.0096 (14)	0.0063 (14)	0.0057 (13)
C18	0.054 (2)	0.087 (3)	0.080 (2)	−0.007 (2)	0.0077 (18)	0.008 (2)
C19	0.080 (2)	0.066 (2)	0.0462 (17)	0.018 (2)	0.0168 (17)	0.0049 (17)
C20	0.0414 (15)	0.0434 (15)	0.0353 (14)	0.0022 (13)	0.0044 (12)	−0.0009 (12)
C21	0.0415 (15)	0.0402 (14)	0.0347 (13)	0.0003 (12)	−0.0029 (12)	0.0028 (12)
C22	0.0377 (14)	0.0422 (15)	0.0400 (14)	−0.0043 (12)	−0.0001 (12)	−0.0015 (12)
C23	0.0424 (15)	0.0470 (15)	0.0381 (14)	−0.0050 (14)	0.0016 (12)	−0.0020 (13)
C24	0.092 (3)	0.057 (2)	0.062 (2)	−0.020 (2)	0.002 (2)	0.0159 (17)
C25	0.0452 (16)	0.0478 (16)	0.0361 (14)	0.0033 (13)	−0.0030 (13)	0.0012 (12)
C26	0.0502 (19)	0.089 (3)	0.057 (2)	0.0166 (19)	−0.0059 (16)	0.0103 (19)

### Geometric parameters (Å, °)

**Table:**

O1—C1	1.425 (4)	C7—H7A	0.9800
O1—C8	1.434 (3)	C8—C9	1.505 (4)
O2—C1	1.419 (4)	C8—H8A	0.9800
O2—C3	1.430 (5)	C9—C10	1.523 (4)
O3—C4	1.421 (4)	C9—H9A	0.9800
O3—C5	1.434 (5)	C10—H10A	0.9800
O4—C4	1.422 (4)	C11—H11A	0.9600
O4—C7	1.429 (3)	C11—H11B	0.9600
O5—C9	1.437 (4)	C11—H11C	0.9600
O5—H5A	0.8200	C12—C13	1.508 (5)
O6—C10	1.407 (4)	C12—H12B	0.9800
O6—C11	1.417 (5)	C13—H13A	0.9600
O7—C10	1.410 (4)	C13—H13B	0.9600
O7—C12	1.437 (4)	C13—H13C	0.9600
O8—C14	1.423 (3)	C14—C15	1.519 (4)
O8—C21	1.441 (3)	C14—C17	1.555 (4)
O9—C14	1.409 (4)	C15—H15A	0.9600
O9—C16	1.433 (4)	C15—H15B	0.9600
O10—C17	1.414 (4)	C15—H15C	0.9600
O10—C18	1.432 (4)	C16—H16A	0.9600
O11—C17	1.424 (3)	C16—H16B	0.9600
O11—C20	1.438 (3)	C16—H16C	0.9600
O12—C22	1.414 (3)	C17—C19	1.520 (4)
O12—H12A	0.8200	C18—H18A	0.9600
O13—C23	1.406 (3)	C18—H18B	0.9600
O13—C24	1.427 (4)	C18—H18C	0.9600
O14—C23	1.415 (4)	C19—H19A	0.9600
O14—C25	1.444 (3)	C19—H19B	0.9600
C1—C2	1.513 (5)	C19—H19C	0.9600
C1—C4	1.555 (5)	C20—C21	1.501 (4)
C2—H2A	0.9600	C20—C25	1.519 (4)
C2—H2B	0.9600	C20—H20A	0.9800
C2—H2C	0.9600	C21—C22	1.514 (4)
C3—H3A	0.9600	C21—H21A	0.9800
C3—H3B	0.9600	C22—C23	1.518 (4)
C3—H3C	0.9600	C22—H22A	0.9800
C4—C6	1.507 (4)	C23—H23A	0.9800
C5—H5B	0.9600	C24—H24A	0.9600
C5—H5C	0.9600	C24—H24B	0.9600
C5—H5D	0.9600	C24—H24C	0.9600
C6—H6A	0.9600	C25—C26	1.522 (4)
C6—H6B	0.9600	C25—H25A	0.9800
C6—H6C	0.9600	C26—H26A	0.9600
C7—C8	1.500 (4)	C26—H26B	0.9600
C7—C12	1.519 (4)	C26—H26C	0.9600
			
C1—O1—C8	111.9 (2)	C7—C12—H12B	109.5
C1—O2—C3	116.8 (3)	C12—C13—H13A	109.5
C4—O3—C5	115.0 (3)	C12—C13—H13B	109.5
C4—O4—C7	112.5 (2)	H13A—C13—H13B	109.5
C9—O5—H5A	109.5	C12—C13—H13C	109.5
C10—O6—C11	114.0 (3)	H13A—C13—H13C	109.5
C10—O7—C12	115.4 (2)	H13B—C13—H13C	109.5
C14—O8—C21	112.0 (2)	O9—C14—O8	110.4 (2)
C14—O9—C16	116.4 (3)	O9—C14—C15	112.5 (2)
C17—O10—C18	116.7 (3)	O8—C14—C15	106.3 (2)
C17—O11—C20	112.7 (2)	O9—C14—C17	104.6 (2)
C22—O12—H12A	109.5	O8—C14—C17	110.4 (2)
C23—O13—C24	113.6 (2)	C15—C14—C17	112.7 (3)
C23—O14—C25	115.5 (2)	C14—C15—H15A	109.5
O2—C1—O1	110.0 (3)	C14—C15—H15B	109.5
O2—C1—C2	113.2 (3)	H15A—C15—H15B	109.5
O1—C1—C2	105.4 (3)	C14—C15—H15C	109.5
O2—C1—C4	103.3 (3)	H15A—C15—H15C	109.5
O1—C1—C4	111.7 (2)	H15B—C15—H15C	109.5
C2—C1—C4	113.4 (3)	O9—C16—H16A	109.5
C1—C2—H2A	109.5	O9—C16—H16B	109.5
C1—C2—H2B	109.5	H16A—C16—H16B	109.5
H2A—C2—H2B	109.5	O9—C16—H16C	109.5
C1—C2—H2C	109.5	H16A—C16—H16C	109.5
H2A—C2—H2C	109.5	H16B—C16—H16C	109.5
H2B—C2—H2C	109.5	O10—C17—O11	110.4 (3)
O2—C3—H3A	109.5	O10—C17—C19	113.2 (3)
O2—C3—H3B	109.5	O11—C17—C19	105.4 (2)
H3A—C3—H3B	109.5	O10—C17—C14	103.8 (2)
O2—C3—H3C	109.5	O11—C17—C14	111.7 (2)
H3A—C3—H3C	109.5	C19—C17—C14	112.6 (3)
H3B—C3—H3C	109.5	O10—C18—H18A	109.5
O3—C4—O4	109.8 (3)	O10—C18—H18B	109.5
O3—C4—C6	112.8 (3)	H18A—C18—H18B	109.5
O4—C4—C6	106.3 (3)	O10—C18—H18C	109.5
O3—C4—C1	104.1 (3)	H18A—C18—H18C	109.5
O4—C4—C1	111.1 (2)	H18B—C18—H18C	109.5
C6—C4—C1	112.9 (3)	C17—C19—H19A	109.5
O3—C5—H5B	109.5	C17—C19—H19B	109.5
O3—C5—H5C	109.5	H19A—C19—H19B	109.5
H5B—C5—H5C	109.5	C17—C19—H19C	109.5
O3—C5—H5D	109.5	H19A—C19—H19C	109.5
H5B—C5—H5D	109.5	H19B—C19—H19C	109.5
H5C—C5—H5D	109.5	O11—C20—C21	109.0 (2)
C4—C6—H6A	109.5	O11—C20—C25	108.0 (2)
C4—C6—H6B	109.5	C21—C20—C25	109.9 (2)
H6A—C6—H6B	109.5	O11—C20—H20A	110.0
C4—C6—H6C	109.5	C21—C20—H20A	110.0
H6A—C6—H6C	109.5	C25—C20—H20A	110.0
H6B—C6—H6C	109.5	O8—C21—C20	109.8 (2)
O4—C7—C8	109.2 (2)	O8—C21—C22	109.8 (2)
O4—C7—C12	108.5 (2)	C20—C21—C22	110.4 (2)
C8—C7—C12	110.4 (2)	O8—C21—H21A	108.9
O4—C7—H7A	109.6	C20—C21—H21A	108.9
C8—C7—H7A	109.6	C22—C21—H21A	108.9
C12—C7—H7A	109.6	O12—C22—C21	112.7 (2)
O1—C8—C9	109.6 (2)	O12—C22—C23	106.3 (2)
O1—C8—C7	109.0 (2)	C21—C22—C23	107.3 (2)
C9—C8—C7	111.5 (2)	O12—C22—H22A	110.2
O1—C8—H8A	108.9	C21—C22—H22A	110.2
C9—C8—H8A	108.9	C23—C22—H22A	110.2
C7—C8—H8A	108.9	O13—C23—O14	111.7 (2)
O5—C9—C8	113.0 (2)	O13—C23—C22	106.8 (2)
O5—C9—C10	106.6 (2)	O14—C23—C22	112.5 (2)
C8—C9—C10	108.1 (2)	O13—C23—H23A	108.6
O5—C9—H9A	109.7	O14—C23—H23A	108.6
C8—C9—H9A	109.7	C22—C23—H23A	108.6
C10—C9—H9A	109.7	O13—C24—H24A	109.5
O7—C10—O6	112.6 (3)	O13—C24—H24B	109.5
O7—C10—C9	112.7 (2)	H24A—C24—H24B	109.5
O6—C10—C9	105.4 (3)	O13—C24—H24C	109.5
O7—C10—H10A	108.6	H24A—C24—H24C	109.5
O6—C10—H10A	108.6	H24B—C24—H24C	109.5
C9—C10—H10A	108.6	O14—C25—C20	108.3 (2)
O6—C11—H11A	109.5	O14—C25—C26	106.1 (2)
O6—C11—H11B	109.5	C20—C25—C26	113.3 (3)
H11A—C11—H11B	109.5	O14—C25—H25A	109.7
O6—C11—H11C	109.5	C20—C25—H25A	109.7
H11A—C11—H11C	109.5	C26—C25—H25A	109.7
H11B—C11—H11C	109.5	C25—C26—H26A	109.5
O7—C12—C13	107.1 (3)	C25—C26—H26B	109.5
O7—C12—C7	108.9 (2)	H26A—C26—H26B	109.5
C13—C12—C7	112.3 (3)	C25—C26—H26C	109.5
O7—C12—H12B	109.5	H26A—C26—H26C	109.5
C13—C12—H12B	109.5	H26B—C26—H26C	109.5

### Hydrogen-bond geometry (Å, °)

**Table:**

*D*—H···*A*	*D*—H	H···*A*	*D*···*A*	*D*—H···*A*
O5—H5A···O13^i^	0.82	2.17	2.907 (3)	150
O12—H12A···O5^ii^	0.82	2.10	2.846 (3)	152

Symmetry codes: (i) −*x*+1, *y*+1/2, −*z*+3/2; (ii) *x*, *y*−1, *z*.
